# Reactivation of Intestinal Inflammation Is Suppressed by Catestatin in a Murine Model of Colitis *via* M1 Macrophages and Not the Gut Microbiota

**DOI:** 10.3389/fimmu.2017.00985

**Published:** 2017-08-21

**Authors:** Mohammad F. Rabbi, Nour Eissa, Peris M. Munyaka, Laëtitia Kermarrec, Omar Elgazzar, Ehsan Khafipour, Charles N. Bernstein, Jean Eric Ghia

**Affiliations:** ^1^Department of Immunology, University of Manitoba, Winnipeg, MB, Canada; ^2^The Children Research Hospital Research Institute of Manitoba, University of Manitoba, Winnipeg, MB, Canada; ^3^Department of Animal Sciences, University of Manitoba, Winnipeg, MB, Canada; ^4^Department of Medical Microbiology, University of Manitoba, Winnipeg, MB, Canada; ^5^Department of Internal Medicine, Section of Gastroenterology, University of Manitoba, Winnipeg, MB, Canada; ^6^Inflammatory Bowel Disease Clinical and Research Centre, University of Manitoba, Winnipeg, MB, Canada

**Keywords:** inflammatory bowel disease, chromogranin-A, catestatin, gut inflammation, M1 and M2 macrophage, gut dysbiosis

## Abstract

While there is growing awareness of a relationship between chromogranin-A (CHGA) and susceptibility to inflammatory conditions, the role of human catestatin [(hCTS); CHGA_352–67_] in the natural history of established inflammatory bowel disease is not known. Recently, using two different experimental models, we demonstrated that hCTS-treated mice develop less severe acute colitis. We have also shown the implication of the macrophages in this effect. The aims of this study were to determine (1) whether hCTS treatment could attenuate the reactivation of inflammation in adult mice with previously established chronic colitis; (2) whether this effect is mediated through macrophages or the gut microbiota. Quiescent colitis was induced in 7–8-week-old C57BL6 mice using four cycles (2–4%) of dextran sulfate sodium. hCTS (1.5 mg/kg/day) treatment or vehicle started 2 days before the last induction of colitis and continuing for 7 days. At sacrifice, macro- and microscopic scores were determined. Colonic pro-inflammatory cytokines [interleukin (IL)-6, IL-1β, and TNF- α], anti-inflammatory cytokines (IL-10, TGF- β), classically activated (M1) (*iNOS, Mcp1*), and alternatively activated (M2) (*Ym1, Arg1*) macrophages markers were studied using ELISA and/or RT-qPCR. *In vitro*, peritoneal macrophages isolated from naïve mice and treated with hCTS (10^−5^ M, 12 h) were exposed to either lipopolysaccharide (100 ng/ml, 12 h) to polarize M1 macrophages or to IL-4/IL-13 (20 ng/ml) to polarize M2 macrophages. M1/M2 macrophage markers along with cytokine gene expression were determined using RT-qPCR. Feces and mucosa-associated microbiota (MAM) samples were collected, and the V4 region of 16 s rRNA was sequenced. Micro- and macroscopic scores, colonic IL-6, IL-1β, TNF- α, and M1 macrophages markers were significantly decreased in the hCTS-treated group. Treatment did not have any effect on colonic IL-10, TGF-β, and M2 markers nor modified the bacterial richness, diversity, or the major phyla in colitic fecal and MAM samples. *In vitro*, pro-inflammatory cytokines levels, as well as their gene expression, were significantly reduced in hCTS-treated M1 macrophages. hCTS treatment did not affect M2 macrophage markers. These findings suggest that hCTS treatment attenuates the severity of inflammatory relapse through the modulation of the M1 macrophages and the release of pro-inflammatory cytokines.

## Introduction

Inflammatory bowel diseases (IBDs) are well-known intestinal disorders that are characterized by chronic relapse and remitting stage of inflammation of gastrointestinal tract (1). Ulcerative colitis (UC) (2) and Crohn’s disease (CD) (3) are two major types of IBD and disease prevalence is rising in the western world (4–6). Currently, the therapeutic drugs available are not curative, and their major goals are to prolong the remission stage by suppressing inflammatory cytokines, such as TNF-α or various corticosteroids and thiopurines (7–9). These drugs require life-long application, are expensive, and can cause adverse effects such as infectious disease and even malignancy because of a dysregulated immune system (10, 11). Moreover, many patients are resistant to these drugs (9, 12, 13). Because of these complications, clinicians have a limited choice of drugs to treat IBD. Therefore, development of a safe, cost-effective curative treatment option for IBD patients is needed.

It has been shown that a complex network of events at the molecular, cellular, and tissue levels underlie inflammation and remodeling that eventually leads to the development of IBD symptoms (14). Mucosal and transmural inflammation is a characteristic feature of IBD that is accompanied by a massive infiltration of activated cells from both the innate and adaptive immune systems (14, 15). In addition to this immune dysregulation, in IBD and experimental colitis (16), gut inflammation is associated with changes in the prohormone chromogranin-A (CHGA)-producing enterochromaffin (EC) cells (17), but the impact of this modification on the immune activation during development of gut inflammation is unclear.

CHGA is a member of the secretory granin protein family, localized at 14q32 of the human genome, and it consists of eight exons and seven introns. It is a prohormone that can be cleaved at its basic residues by prohormone convertases 1/3 or 2, carboxypeptidase E/H22 to generate shorter bioactive peptides (18, 19). These bioactive peptides derived from the C and N terminus are highly conserved among vertebrates, up to 88% of sequence homology between species (20–22). CHGA-derived peptides (CGDPs) regulate several physiological activities and the immune system (23–25). One of the most important biologically active peptides derived from the conserved C-terminal region of CHGA is the antihypertensive peptide catestatin human catestatin [(hCTS); hCHGA_352–372_] (26–28). hCTS is cationic in nature and is well known for its *in vitro* antimicrobial properties, it possesses immune regulation properties (29), and it can stimulate chemotaxis of human peripheral blood monocytes (23).

Recently, our group and others confirmed that serum CHGA levels are elevated in patients with UC (30, 31). Moreover, CHGA demonstrated a positive correlation with inflammatory markers, such as procalcitonin, C-reactive protein, and simplified acute physiological score (32). Taken together, all these data provide a proof of the concept that CHGA and CGDPs play an important role in the development of inflammatory pathologies, but the knowledge of pathophysiological mechanisms whereby CGDPs exert their action remains unclear, especially in the context of gut inflammation. Consequently, the link between CGDPs and colitis remains to be confirmed. Our lab recently showed a preventive effect of hCTS on gut inflammation using two acute experimental murine models of colitis (31). Historically, UC patients have flares of inflammation and relapse in between, which cannot be mimicked using the acute model of colitis. Therefore, we delineated the effect of hCTS on chronic gut inflammation using the quiescence colitis model.

Macrophages are innate immune cells and are instrumental in protecting the host from the luminal content of intestinal tract (33, 34). Depending on the stimuli, these cells can be polarized to classically activated (M1) and alternatively activated (M2) macrophages (35). Interferon-gamma (IFNγ) and lipopolysaccharide (LPS) are mainly responsible for the M1 polarization and, in return, M1 macrophages release or generate nitric oxide (NO) and other pro-inflammatory cytokines, such as interleukin (IL)-6, IL-1β, and TNF-α to initiate immune activation (35, 36). Conversely, the M2 macrophages are activated upon stimulation by IL 4, 13, and can subsequently downregulate the inflammatory process through the production of IL10, polyamines, and prolines (37). During UC and experimental colitis, M1 macrophages infiltrate the intestinal tissue (38), but this immune activation can be counter-balanced by the M2 macrophage population (38).

Beside immune cells, the gastrointestinal tract is heavily colonized with the microbial population. On average, 10^14^ microbes representing 1,000 species are present within the human gastrointestinal tract (39). In healthy individuals, microbial diversity in the intestine is stable over time and has a symbiotic relationship with the host, which is crucial to maintain a healthy gut immune system (40). For example, gut microbiota can activate toll-like receptors (TLRs) in the gut epithelium, which in turn can affect the expression of antimicrobial peptides such as angiogenins (41, 42). In addition to the innate immune system and the macrophages, gut microbiota can also control the host’s adaptive immune system through T cell receptor αβ-positive intraepithelial lymphocytes, T regulatory cells, and T helper 17 (43). It has been observed that microbial diversity is altered both in animal models of colitis and in UC patients. In our recent study, we observed that acute exposure of dextran sulfate sodium (DSS) can alter the murine gut microbiota by reducing the abundance of Bacteroides compared to Firmicutes (44). Moreover, we showed that hCTS treatment could alter the gut microbial composition in the naïve mice (45).

The aim of our study was to demonstrate the effect of hCTS on chronic gut inflammation. Using a model of quiescence/reactivation colitis, we determined the effect of the hCTS on colitic inflammatory markers, macrophage population, and gut microbiota. We demonstrated that intrarectal administration of hCTS downregulates the inflammatory process through downregulation of the pro-inflammatory properties of the M1 macrophage population, but not microbiota.

## Materials and Methods

### Animals

Male C57BL/6 mice (7–9 weeks old) were purchased from Charles River (Canada) and maintained in the animal care facility at the University of Manitoba. The experimental protocol was approved by the University of Manitoba Animal Ethics Committee (15-010) and the research was conducted according to the Canadian Guidelines for Animal Research (46, 47).

### Peptide

The hCTS (hCHGA_352–372_: SSMKLSFRARAYGFRGPGPQL) was used (Biopeptide Co., Inc., San Diego, CA, USA), and the peptide was injected intrarectally (*i.r*.) at 1.5 mg/per kg body weight per day for 7 days. Saline (0.9%) was injected into the control group. Mice were anesthetized using Isoflurane (Abbott, Toronto, ON, Canada). PE-90 tubing (10 cm long; ClayAdam, Parsippany, NJ, USA), which was attached to a tuberculin syringe (BD, Mississauga, ON, Canada), was inserted 3.5 cm into the colon. The dose was determined according to our previous published study (31).

### Quiescence DSS Colitis

To develop quiescence colitis, recurrent cycles of DSS was administered in mice according to a protocol by *Perse* M and Cerar A (48) with some modification. In summary, four cycles of DSS (molecular weight, 40 kDa: MPI Biomedicals, Santa Ana, CA, USA) at concentrations of 4, 2, 2, and 4% (weight/volume; drinking water) were performed. Each DSS cycle lasted for 5 days and was followed by 11 days of rest (access to normal drinking water) and treatment started 2 days before the reactivation.

### Determination of Colitis Severity Disease Activity Index (DAI)

The colitis severity DAI is a composite score of weight loss, stool consistency and bleeding (49), which was assessed on a daily basis. Scores were defined as follows: weight: 0, no loss; 1, 5–10%; 2, 10–15%; 3, 15–20%; and 4, 20% weight loss; stool: 0, normal; 2, loose stool; and 4, diarrhea; and bleeding: 0, no blood; 2, presence of blood; and 4, gross blood. Blood was assessed using the Hemoccult II test (Beckman Coulter, Oakville, ON, Canada).

### Sample Collection

Following the final reactivation, animals were euthanized under isoflurane (50) anesthesia and samples were collected. The macroscopic score was determined based on stool consistency, rectal prolapse, and rectal and colonic bleeding (49). A fecal sample (250 mg) and a portion of the colon were collected in individual collector tubes from each animal.

### Microscopic Score and Colonic Cytokines

The colonic histology (microscopic score) was assessed by hematoxylin-eosin (H&E) (Sigma, Mississauga, ON, Canada) staining of 3-µm formalin (Sigma, Mississauga, ON, Canada)-fixed colon sections. A scoring system composed of architectural derangements, goblet cell depletion, edema/ulceration, and degree of inflammatory cell infiltrate was used to assess the colonic damage (49). To assess colonic cytokine levels, distal colon samples were homogenized in 700 µl of Tris–HCl buffer containing protease inhibitors (Sigma, Mississauga, ON, Canada) and then centrifuged for 30 min at 4°C. Then, the supernatant was frozen at −80°C until assay. Commercial ELISA kits were used to determine cytokine levels (IL-1β, IL-6, TNF-α, IL-10, and TGF-β) (R&D Systems, Minneapolis, MN, USA), according to the manufacturer’s instructions.

### Macrophage Isolation

Naïve peritoneal macrophages were collected as described by Rabbi et al. (31). Briefly, sterile PBS (Gibco BRL Life Technologies, Grand Island, NY, USA) (10 ml) was injected into the caudal half of the peritoneal cavity using a 25-gage needle (BD, Mississauga, ON, Canada) and the body was shaken for 15 s. Resident peritoneal cells were slowly withdrawn using a 19-gage needle (BD, Mississauga, ON, Canada), and they were purified by adhesion. Resident peritoneal cells were plated in macrophage culture medium [RPMI 1640 supplemented with 10% HI FCS containing 50 IU of penicillin, 50 µg streptomycin, and 2 mM glutamine per milliliter (Gibco BRL Life Technologies, Grand Island, New York, NY, USA)] for 60 min at 37°C. Non-adherent cells were removed by washing five times with 500 µl of warm PBS. Overall cell viability of the adherent cell before and after treatment was greater than 97%, and more than 94% of the cells were macrophages using DiffQuick (Jorgensen Laboratory, Loveland, CO, USA) staining. Cells were maintained at 37°C in a humidified incubator containing 5% CO_2_. M1 macrophages were stimulated by adding LPS (Sigma, Mississauga, ON, Canada) to the cultures at a final concentration of 100 ng/ml. M2 macrophages were obtained by adding IL4/IL13 combination (20 ng/ml). hCTS was added to the medium at a final concentration of 10^−5^ M 1 h before the LPS or IL4/IL13. Supernatants were collected 24 h after LPS or IL4/IL13. For gene expression analysis, 1 ml of Trizol solution was added per well to collect mRNA from the cells, which were stored in −0°C until further use.

### Gene Expression Analysis

Total RNA was extracted using a kit (TRIzol^®^ Plus RNA Purification Kit, Life Technologies, New York, NY, USA), according to the manufacturer’s instructions. Quality and quantity of RNA were determined by measuring the absorbance at 260 and 280 nm using NanoDrop ND-1000 UV-Vis Spectrophotometer (Thermo Fisher Scientific, Waltham, MA, USA). All samples absorption ratio (A260/A280) ranged between 1.8 and 2.2 (51, 52). To remove genomic DNA contamination, samples were treated with RQ1 RNase-Free DNase^®^ (Promega Corporation, Madison, WI, USA), according to the manufacturer’s instructions. Reverse transcription was performed using SuperScript VILO cDNA Synthesis Master Mix (Invitrogen, Grand Island, New York, NY, USA), according to the manufacturer’s instructions. qReal-time PCRs (qRT-PCRs) were performed in a Roch lightCycler 96 Real-Time System using Power SYBR green master mix (Life Technologies) in a final volume of 20 µl reactions. All samples were tested in triplicate. Differences in the threshold cycle (ΔCt) number were determined between the target genes and the housekeeping gene *Eukaryotic Elongation factor 2* (*Eef2*) (51), which was used to calculate differences in expression. The primers were designed from nucleotide sequences identified using NCBI BLAST (http://blast.ncbi.nlm.nih.gov/Blast.cgi) to confirm the specificity of the primer design. The primer characteristics of nominated genes are listed in Table 1.

**Table 1:** RT-qPCR primers sequences.

Gene	Forward	Reverse
*Eef2*	TGTCAGTCATCGCCCATGTG	CATCCTTGCGAGTGTCAGTGA
*iNOS*	GTTCTCAGCCCAACAATACAAGA	GTGGACGGGTCGATGTCAC
*Il1b*	GCAACTGTTCCTGAACTCAACT	ATCTTTTGGGGTCCGTCAACT
*Il6*	TAGTCCTTCCTACCCCAATTTCC	TTGGTCCTTAGCCACTCCTTC
*Tnfa*	CCCTCACACTCAGATCATCTTCT	GCTACGACGTGGGCTACAG
*Mcp1*	TTAAAAACCTGGATCGGAACCAA	GCATTAGCTTCAGATTTACGGGT
*Il10*	GCTCTTACTGACTGGCATGAG	CGCAGCTCTAGGAGCATGTG
*Ym1*	CAGGTCTGGCAATTCTTCTGAA	GTCTTGCTCATGTGTGTAAGTGA
*Arg*	TTGGGTGGATGCTCACACTG	GTACACGATGTCTTTGGCAGA
*Tgfb*	CTCCCGTGGCTTCTAGTGC	GCCTTAGTTTGGACAGGATCTG

### Illumina Sequencing

DNA was extracted from 250 mg fecal mass and colon mucosa scrapings using a ZR fecal and tissue DNA Kit (Zymo Research Corp., Orange, CA, USA). Genomic DNA was normalized to achieve a concentration of 20 ng/µL and quality-checked by 16S rRNA gene using PCR amplification of 27F (5′-GAAGAGTTTGATCATGGCTCAG-3′) and 342R (5′-CTGCTGCCTCCCGTAG-3′) (53, 54). Amplicons were verified by agarose gel electrophoresis. Library construction and Illumina sequencing were performed as described by Derakhshani et al. (55) targeting the V4 region of the 16S rRNA and using the MiSeq platform (Illumina, San Diego, CA, USA) at the Gut Microbiome and Large Animal Biosecurity Laboratories, Department of Animal Science, University of Manitoba, Canada.

### Bioinformatics Analyses

Bioinformatics analyses were performed as described by Derakhshani et al. (55). Briefly, the PANDAseq assembler (56) was used to merge overlapping paired-end Illumina fastq files. All the sequences with mismatches or ambiguous calls in the overlapping region were discarded. The output fastq file was then analyzed using downstream computational pipelines in the open source software package QIIME (57). Assembled reads were demultiplexed according to the barcode sequences and chimeric reads were filtered using UCHIME (58). Sequences were assigned to operational taxonomic units (OTU) using the QIIME implementation of UCLUST (59) at the 97% pairwise identity threshold. Taxonomies were assigned to the representative sequence of each OTU using an RDP classifier (60) and aligned with the Greengene score reference database (61) using PyNAST algorithms (62). The phylogenetic tree was built with FastTree 2.1.3 (63) for additional comparisons between microbial communities.

### Alpha (α)- and Beta (β)-Diversity Analyses

Within-community diversity (α-diversity) was calculated using QIIME. Rarefaction curves were generated using Chao 1, an estimator of species richness (64) with 10 sampling repetitions at each sampling depth. An even depth of 25,000 and 5,000 sequences per sample was used for calculation of species richness in fecal and colon samples, respectively. To compare microbial composition between samples, β-diversity was measured by calculating the weighted and unweighted Unifrac distances (65) using QIIME default scripts. Principal coordinate analysis was applied on the resulting distance matrices to generate two-dimensional plots using PRIMER v6 software (66). Permutational multivariate analysis of variance of Bray–Curtis distance (67) was used to calculate *P*-values and test for significant differences in β-diversity among treatment groups. α-Diversity differences between treatment groups were determined using SAS (SAS 9.3, 2012).

### Statistical Analysis

All the results are presented as the mean ± SEM. The student’s *t*-test and one-way ANOVA followed by the Bonferroni multiple comparison *post hoc* analysis was used to compare the significance between control and experimental groups. A *P*-value of <0.05 was considered significant, with *n* = 5 to 12 depending on the groups tested (Prism 5, GraphPad, La Jolla, CA, USA). The SAS UNIVARIATE procedure (SAS 9.3, 2012) was used to test the normality of residuals for α biodiversity data. Non-normally distributed data were log transformed and then used to assess the effect of sampling using the SAS MIXED procedure. The SAS MIXED procedure was used, as described above, to test for significant changes in the proportions of different phyla among the groups of interests. All the phyla were divided into two groups of abundant (above 1% of the community) and low-abundance (below 1% of the community). The differences between groups were considered significant at *P* < 0.05 while trends were observed at *P* < 0.1.

## Results

### Effect of hCTS on Weight Loss and Macroscopic Score

**Figure 1:**
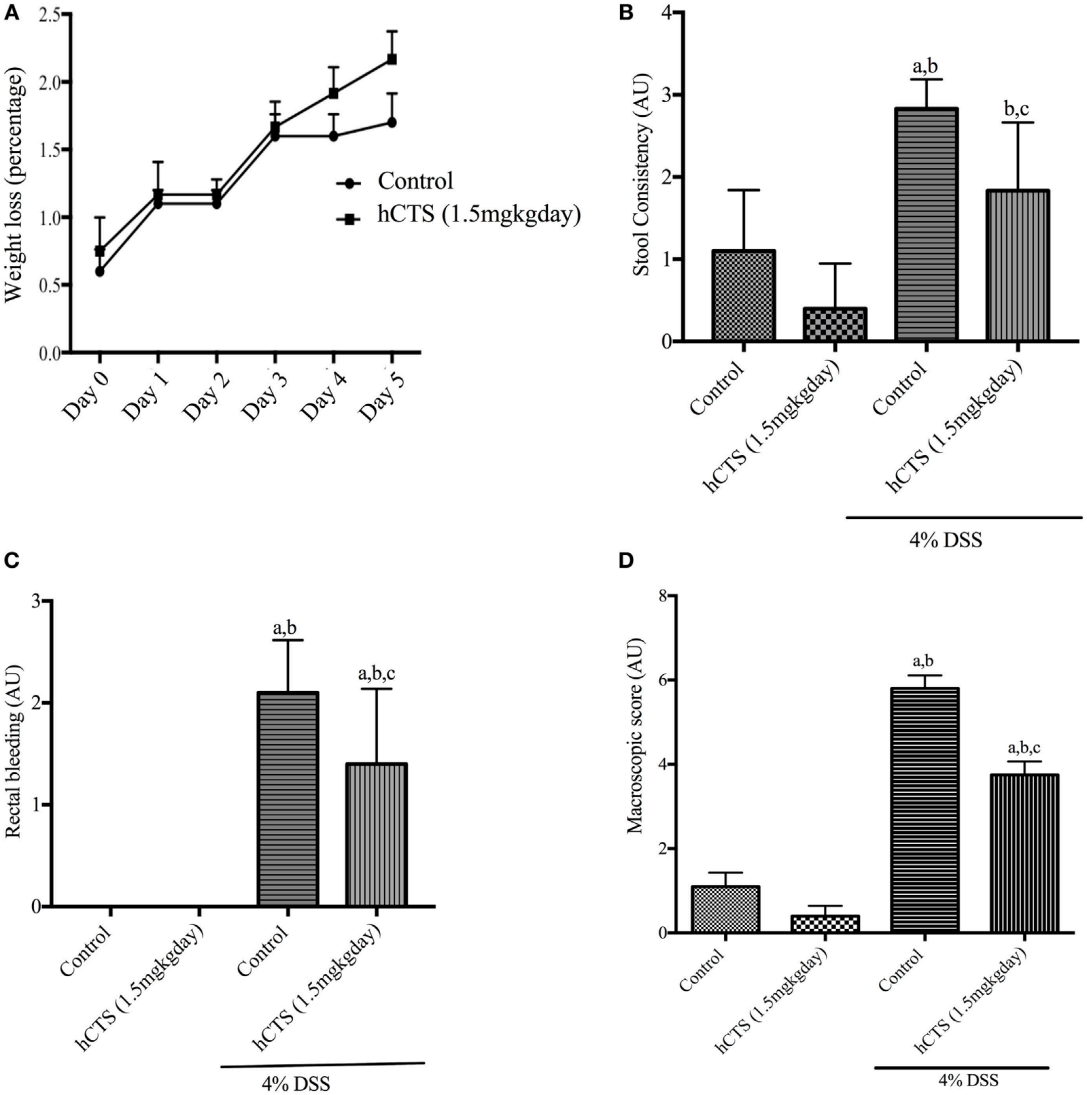
Effect of human catestatin (hCTS) treatment (1.5 mg/kg/day, *i.r*. 7 days) on weight loss and macroscopic score during colitis reactivation in quiescent colitic mice. The 7-day hCTS treatment did not improve weight loss observed in chronic quiescence colitic mice **(A)**. However, during sacrifice hCTS treatment significantly improved stool consistency **(B)**, rectal bleeding **(C)**, and macroscopic score **(D)** in colitic mice. The macroscopic score was determined on the day of sacrifice considering fecal consistency, hyperplasia, and rectal bleeding. AU, arbitrary units, ^a^*P* < 0.05 compared to the Control [No dextran sulfate sodium (DSS)/No hCTS] group; ^b^*P* < 0.05 compared to the hCTS (No DSS) group; ^c^*P* < 0.05 compared to the DSS (No hCTS) group; Bonferroni, one-way ANOVA. Control (*n* = 5), hCTS (*n* = 5), 4% DSS (*n* = 10), 4% DSS + hCTS (*n* = 12).

Human catestatin treatment (1.5 mg/kg/day, 7 days, *i.r*.) did not improve weight loss following reactivation of quiescent colitis (Figure 1A). DAI was also not affected by the treatment (data not shown). At sacrifice, the macroscopic score was assessed by composite scoring of fecal consistency, hyperplasia, and rectal bleeding (49), and hCTS treatment significantly improved stool consistency (Figure 1B), rectal bleeding (Figure 1C) and the macroscopic score when compared with vehicle-treated colitic mice (Figure 1D).

### Effect of hCTS on Microscopic Score

**Figure 2:**
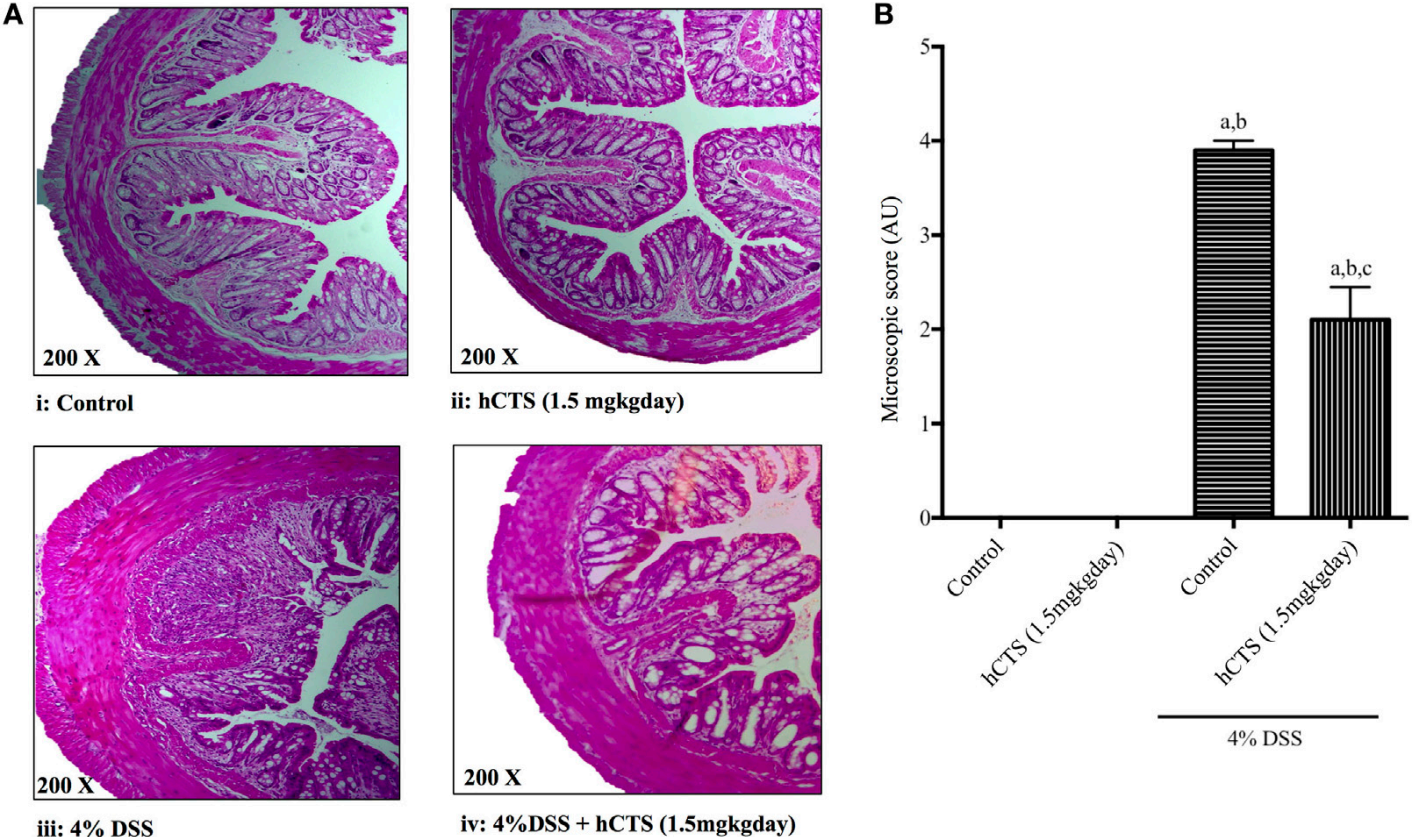
Effect of human catestatin (hCTS) treatment (1.5 mg/kg/day, *i.r*. 7 days) on **(A)** representative colonic architecture and **(B)** composite histological score. When compared to the colonic appearance of non-colitic mice [no hCTS, **(A)**:i and with hCTS, **(A)**:ii], dextran sulfate sodium (DSS) treatment **(A)**:iii resulted in massive infiltration of immune cells and damage of intestinal architecture. This cell infiltration was inhibited together with improvement of tissue architecture in hCTS-treated DSS mice **(A)**:iv. Thus, the composite histological (microscopic) score was significantly improved in hCTS-treated colitic mice compared with vehicle-treated colitic mice **(B)**. AU, arbitrary units; ^a^*P* < 0.05 compared to the Control (No DSS/No hCTS) group; ^b^*P* < 0.05 compared to the hCTS (No DSS) group; ^c^*P* < 0.05 compared to the DSS (No hCTS) group; Bonferroni, one-way ANOVA. Control (*n* = 5), hCTS (*n* = 5), 4% DSS (*n* = 10), 4% DSS + hCTS (*n* = 12).

Colitis severity was assessed in terms of mucosal inflammation and cellular infiltration *via* microscopic scoring of H&E-stained formalin-fixed tissue samples (49). As shown in Figure 2A, DSS treatment resulted in massive tissue infiltration of cells and architectural damage. Following reactivation of quiescent colitis, hCTS treatment (1.5 mg/kg/day, 7 days, *i.r*.) significantly improved tissue architecture, edema, and decreased the cell infiltrate (Figures 2A,B).

### Effect of hCTS on Colonic Cytokine Level

**Figure 3:**
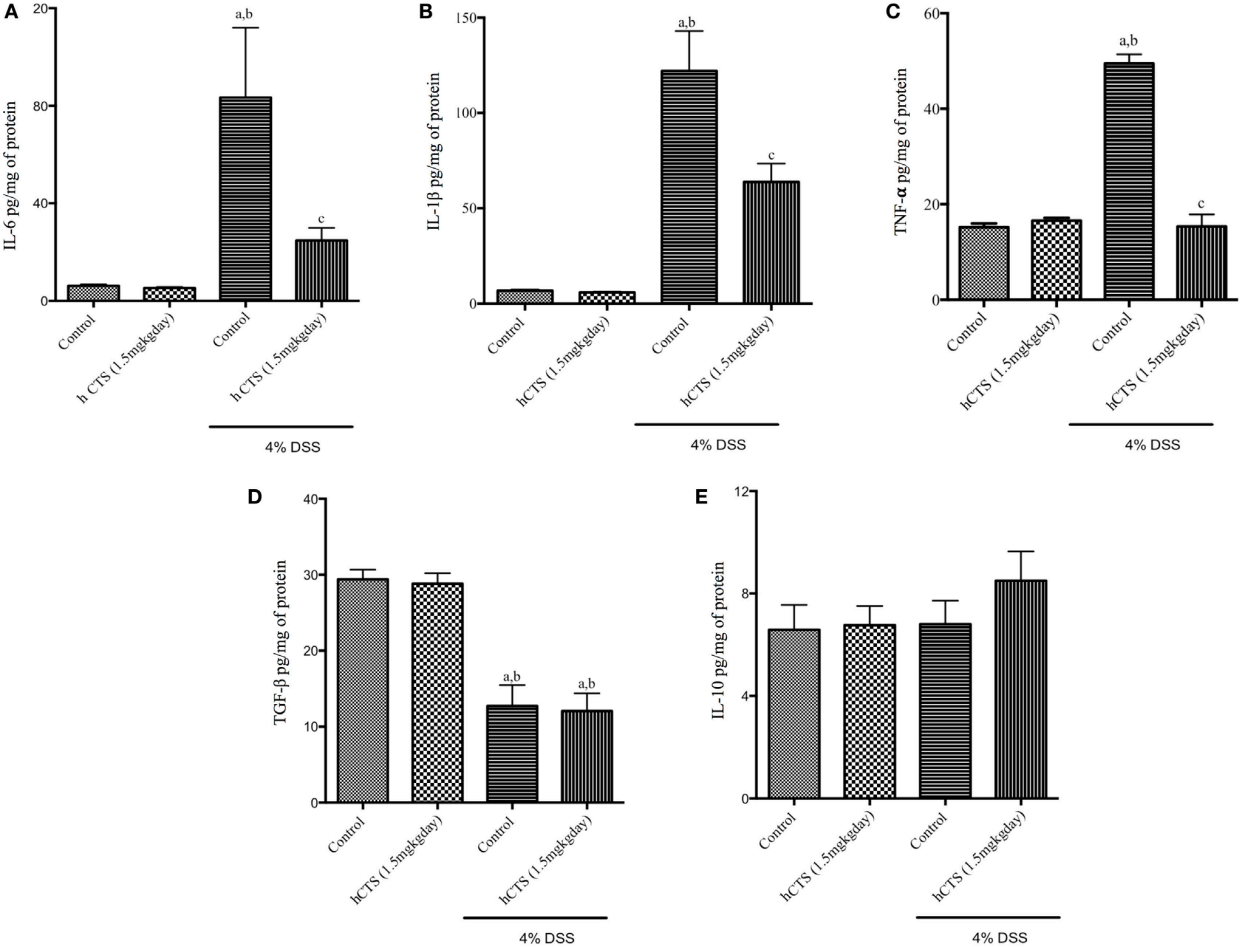
Effect of human catestatin (hCTS) treatment (1.5 mg/kg/day, *i.r*. 7 days) on colonic cytokines. hCTS treatment resulted in significant downregulation of colonic pro-inflammatory cytokines during colitis activation in quiescence colitic mice, including interleukin (IL)-6 **(A)**, IL-1β **(B)**, and TNF- α **(C)**. There was no observable effect of hCTS on colonic anti-inflammatory cytokines in these mice, including TGF- β **(D)** and IL-10 **(E)**. *n* ≥ 5; ^a^*P* < 0.05 compared to the Control [No dextran sulfate sodium (DSS)/No hCTS] group; ^b^*P* < 0.05 compared to the hCTS (No DSS) group; ^c^*P* < 0.05 compared to the DSS (No hCTS) group; Bonferroni, one-way ANOVA. Control (*n* = 5), hCTS (*n* = 5), 4% DSS (*n* = 10), 4% DSS + hCTS (*n* = 12).

To assess the severity of colitis, levels of the major pro-inflammatory cytokines IL-6, IL-1β, and TNF- α and the anti-inflammatory cytokines IL-10 and TGF-β were determined. We confirmed that reactivation of colitis significantly increased colonic level of pro-inflammatory cytokines IL-6, IL-1β, and TNF- α (Figures 3A–C) and decreased level of colonic anti-inflammatory cytokine TGF-β (Figure 3D), but IL-10 levels were not affected (Figure 3E). hCTS treatment (1.5 mg/kg/day, 7 days, *i.r*.) significantly reduced colonic pro-inflammatory cytokines following reactivation of quiescence colitis (Figures 3A–C), but treatment did not improve colonic anti-inflammatory cytokines (Figures 3D,E).

### Effect of hCTS on Colonic M1 and M2 Macrophages Markers/Mediators

**Figure 4:**
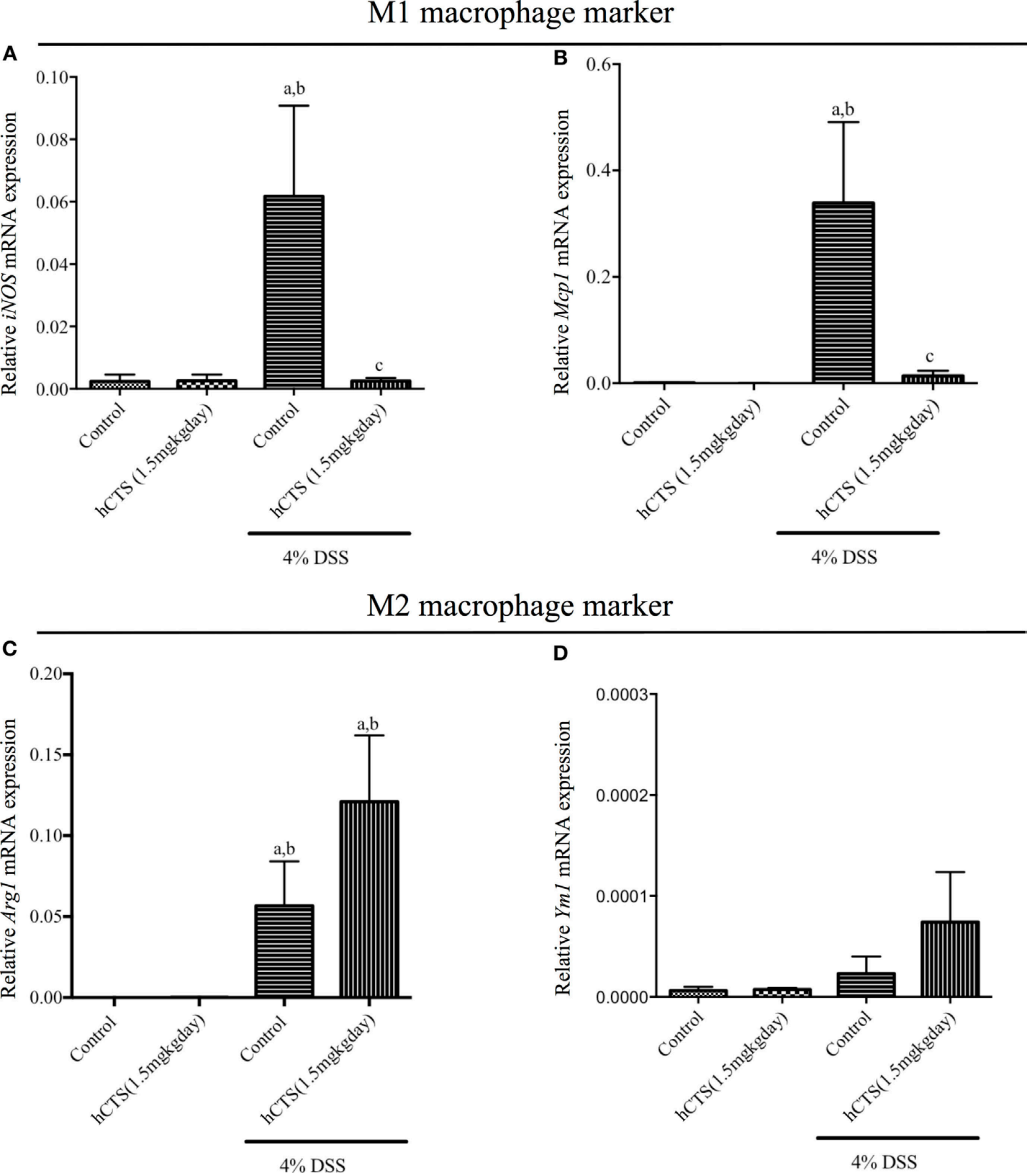
Effect of human catestatin (hCTS) treatment (1.5 mg/kg/day, *i.r*. 7 days) on M1 and M2 macrophage population. hCTS treatment down regulated the M1 macrophage markers in colitic mice, including *inducible nitric oxide synthase* (*iNOS*) **(A)** and monocyte chemoattractant protein-1 (*Mcp1*) **(B)**. However, the effect was not observed in M2 macrophage markers, including arginase 1 (*Arg1*) **(C)** and chitinase-like 3 (*Ym1*) **(D)**. *n* ≥ 5; ^a^*P* < 0.05 compared to the Control [No dextran sulfate sodium (DSS)/No hCTS] group; ^b^*P* < 0.05 compared to the hCTS (No DSS) group; ^c^*P* < 0.05 compared to the DSS (No hCTS) group; Bonferroni, one-way ANOVA. Control (*n* = 5), hCTS (*n* = 5), 4% DSS (*n* = 10), 4% DSS + hCTS (*n* = 12).

Monocytes and macrophages regulate gut inflammation and the majority of tissue infiltrating cells following reactivation of quiescence colitis are M1 macrophage and are the main source of IL-6, IL-1β, and TNF- α (38). To determine the mechanistic anti-inflammatory effect observed after hCTS treatment, mRNA expression of both M1 and M2 macrophage marker/mediators were determined. Reactivation of quiescent colitis was associated with a significant increase of M1 macrophage markers [inducible nitric oxide synthase (*iNOS*) and monocyte chemoattractant protein-1 (*Mcp1*)], which was significantly downregulated by hCTS treatment (Figures 4A,B). Reactivation of quiescent colitis was associated with a significant increase in an M2 macrophage marker [Arginase 1 (*Arg1*)], but not Chitinase-like 3 (*Ym1*) expression (Figures 4C,D), and hCTS treatment (1.5 mg/kg/day, 7 days, *i.r*.) did not modify the markers’ expression (Figures 4C,D).

### Effect of hCTS *In Vitro* Treatment on M1 and M2 Macrophage Function

**Figure 5:**
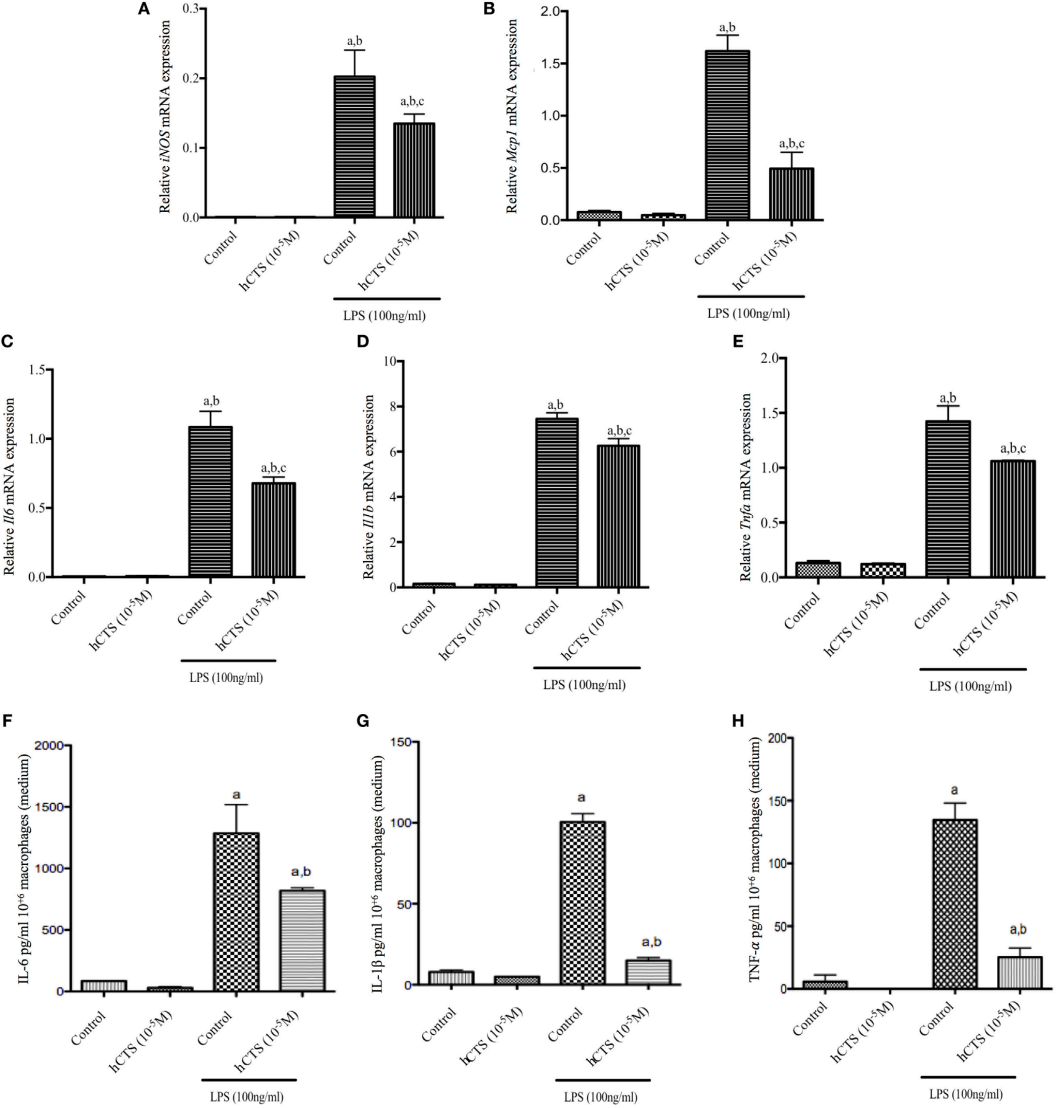
Effect of human catestatin (hCTS) treatment (1.5 mg/kg/day, *i.r*. 7 days) on M1 macrophage population *in vitro*. hCTS treatment resulted in downregulation of M1 macrophage markers and pro-inflammatory cytokine gene expression, including *iNOS* **(A)**, *Mcp1* **(B)**, *Il6* **(C)**, *Il1b* **(D)**, and *Tnfa* **(E)**. The pro-inflammatory cytokine level in the supernatant was also decreased when CTS was present in the medium. Pro-inflammatory cytokines include interleukin (IL)-6 **(F)**, IL-1β **(G)**, and TNF-α **(H)**. *n* = 3; ^a^*P* < 0.05 compared to the medium [no lipopolysaccharide (LPS)]-treated group; ^b^*P* < 0.05 compared to the hCTS (no LPS)-treated group; ^c^*P* < 0.05 compared to the medium (LPS)-treated group; Bonferroni, one-way ANOVA.

**Figure 6:**
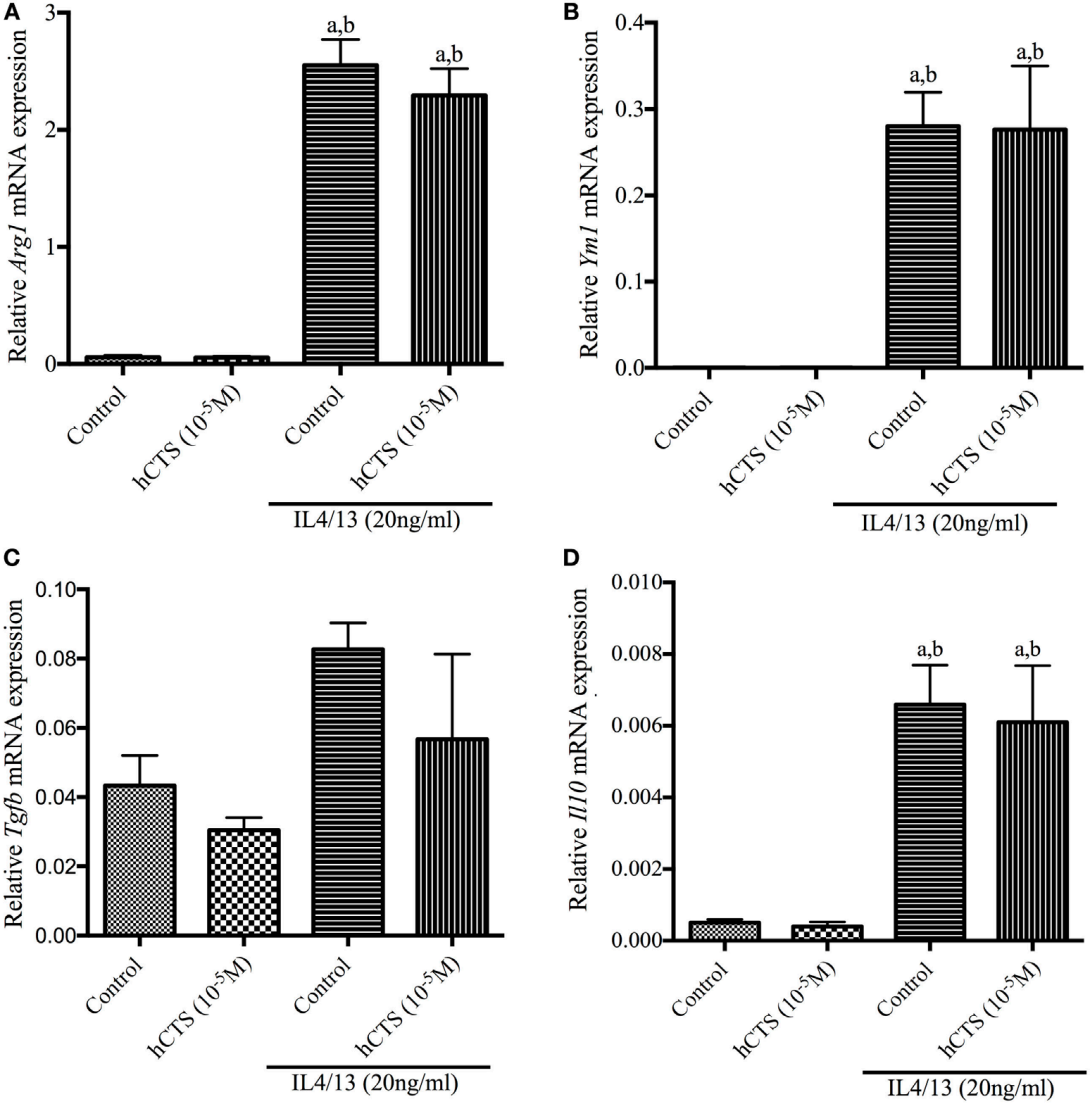
Effect of human catestatin (hCTS) treatment (1.5 mg/kg/day, *i.r*. 7 days) on M2 macrophage population *in vitro*. hCTS treatment did not change gene expression of either M2 macrophage markers or the anti-inflammatory cytokines, including *arginase 1* (*Arg1*) **(A)**; chitinase-like 3 (*Ym1*) **(B)**; *Tgfb* **(C)**; and *Il10* **(D)**. *n* = 3; ^a^*P* < 0.05 compared to the medium [no interleukin (IL)-4/13]-treated group, ^b^*P* < 0.05 compared to the hCTS (no IL-4/13)-treated group; Bonferroni, one-way ANOVA.

Like our chronic quiescence reactivation of colitis hCTS treatment where M1 macrophage marker expression was reduced, we investigated *in vitro* the effect of this peptide on M1 macrophage. In naïve peritoneal macrophages, polarized to an M1 macrophage phenotype, M1 macrophage marker (*iNOS, Mcp1*) and pro-inflammatory cytokine (*Il6, Il1b* and *Tnfa*) gene expression increased (Figures 5A–E). Level of pro-inflammatory cytokines in the supernatant confirmed that increase (Figures 5F–H). Addition of hCTS (10^−5^M) in media 1 h before LPS simulation significantly abrogated the increase in gene expression for M1 macrophage markers and pro-inflammatory cytokines and their secretion in the supernatant (Figures 5A–H). Conversely, hCTS (10^−5^M) treatment neither affected the expression of M2 macrophages markers *Arg1* and *Ym1* (Figures 6A,B) nor the expression of anti-inflammatory cytokines *Tgfb* and *Il10* (Figures 6C,D).

### Effect of hCTS on Gut Microbiota

**Figure 7:**
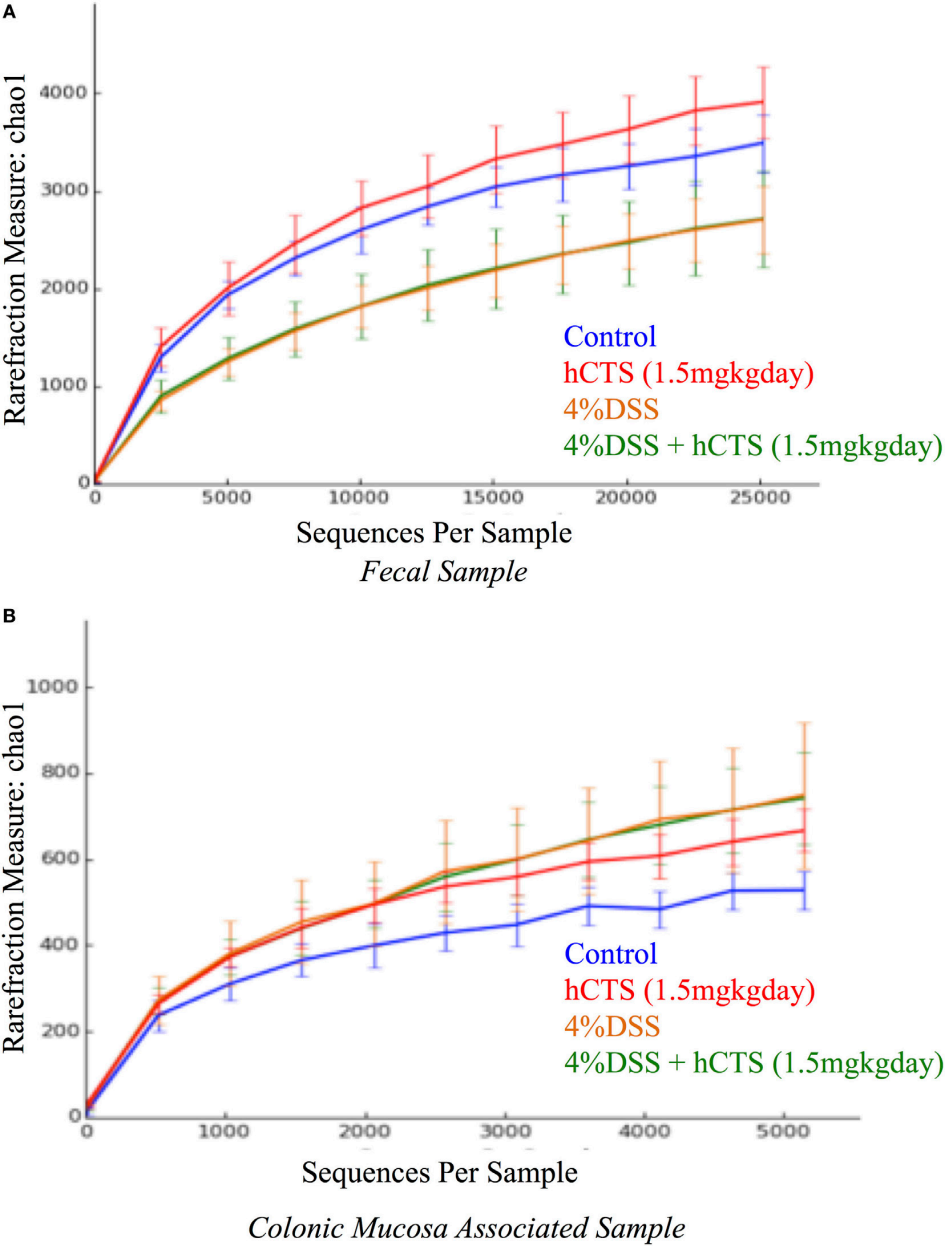
Rarefaction analysis on Chao 1, a measure of species richness based on operational taxonomic unit (OTU). Fecal samples **(A)**; Colonic mucosa-associated microbiota samples **(B)**. Dextran sulfate sodium (DSS) treatment significantly changed microbial diversity compared to non-colitic animals in both sample types. However, the human catestatin (hCTS) treatment (1.5 mg/kg/day, *i.r*. 7 days) did not change the diversity observed in DSS mice. Control (*n* = 2, fecal samples; *n* = 3, colonic mucosa-associated samples), hCTS (*n* = 5), 4% DSS (*n* = 10), 4% DSS + hCTS (*n* = 11, fecal samples; *n* = 12, colonic mucosa-associated samples).

**Figure 8:**
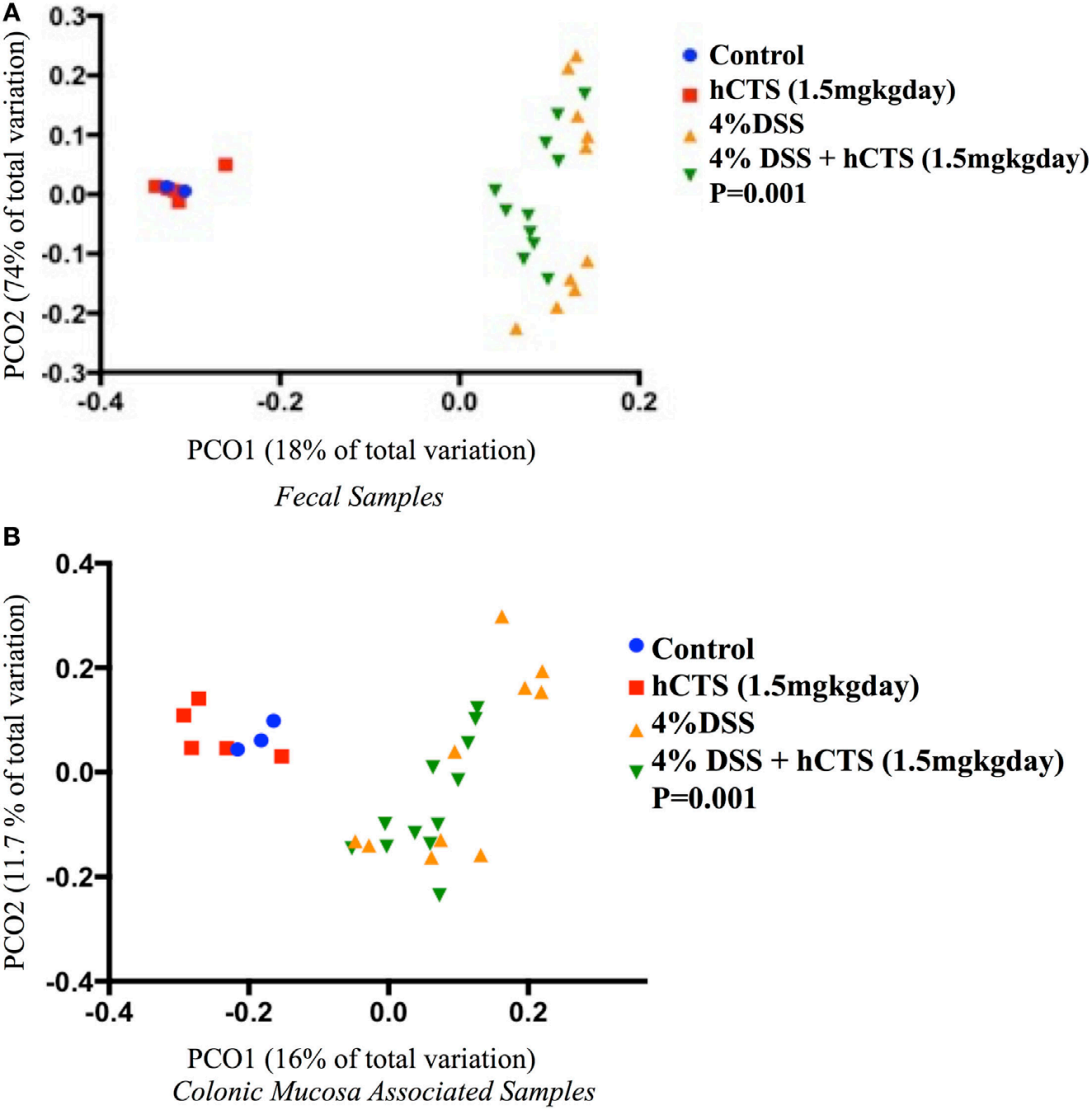
Principal coordinate analysis based on the unweighted UniFrac distance metric. Fecal samples **(A)**; Colonic mucosa-associated microbiota samples **(B)**. Each color point represents an animal and color is shaded according to the treatment. The colitic induction clustered the microbial composition separately compared to non-colitic mice (β diversity, *P*-values <0.05). The human catestatin (hCTS) treatment (1.5 mg/kg/day, *i.r*. 7 days) did not change the microbial composition of colitic mice. *P*-values were calculated using permutational multivariate analysis of variance. Control (*n* = 2, fecal samples; *n* = 3, colonic mucosa-associated samples), hCTS (*n* = 5), 4% dextran sulfate sodium (DSS) (*n* = 10), 4% DSS + hCTS (*n* = 11, fecal samples; *n* = 12, colonic mucosa-associated samples).

**Figure 9:**
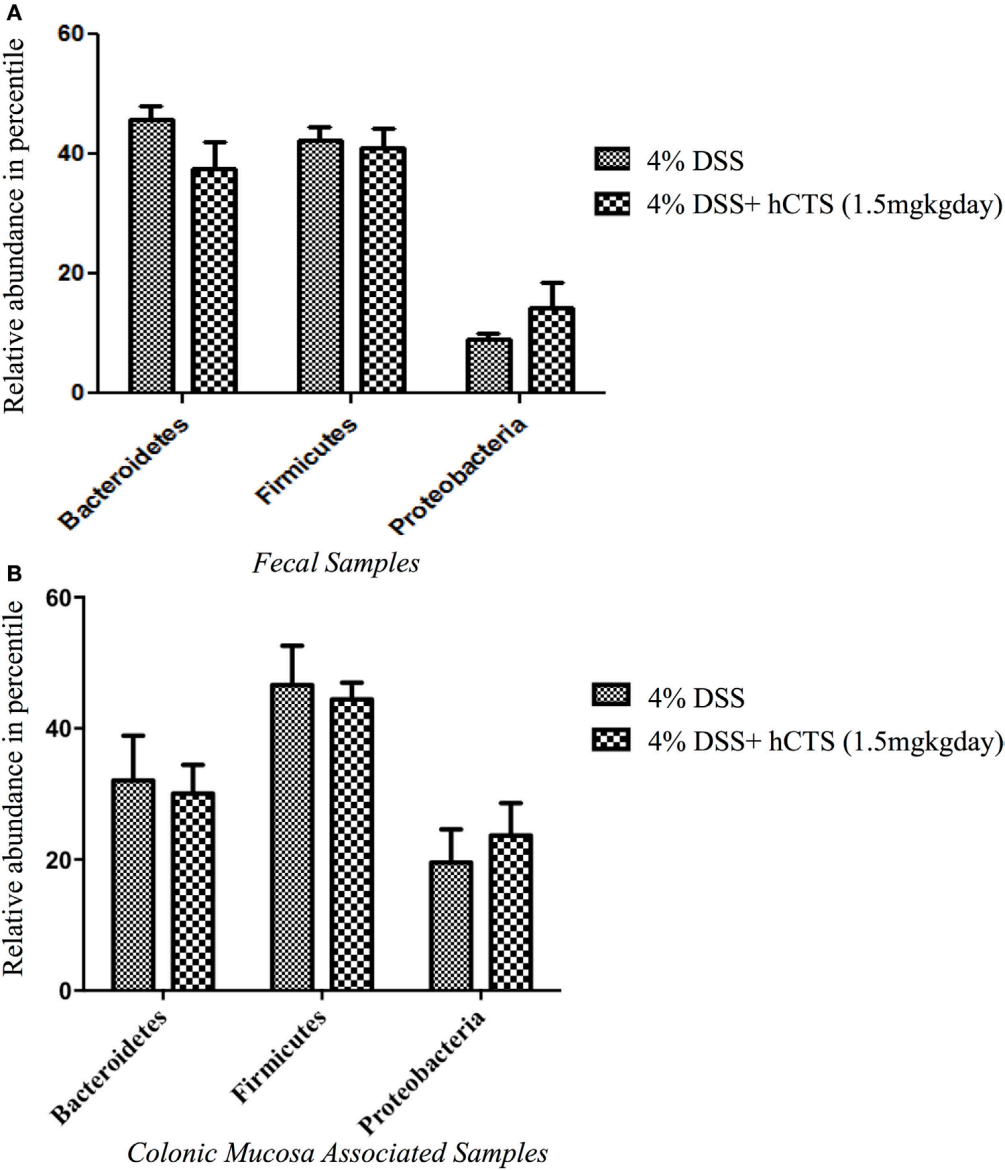
The effect of human catestatin (hCTS) treatment (1.5 mg/kg/day, *i.r*. 7 days) on the abundant phyla (≥1%) present in the fecal **(A)** and colonic mucosa-associated samples **(B)**. The hCTS treatment showed no significant effect on abundant phyla during colitis reactivation in quiescence colitic mice. 4% dextran sulfate sodium (DSS) (*n* = 10), 4% DSS + hCTS (*n* = 11, fecal samples; *n* = 12, colonic mucosa-associated samples).

Gut microbial dysbiosis is evident in both UC and experimental colitis mice (44, 68). Because hCTS is a cationic peptide and well known as an antimicrobial peptide *in vitro*, we investigated the effect of this peptide on gut microbiota. Bacterial richness and diversity from both fecal and colonic mucosa-associated samples were determined. Following reactivation of quiescent colitis, bacterial species richness in both fecal and colonic mucosa-associated samples were modified (Figures 7A,B). However, hCTS treatment (1.5 mg/kg/day, 7 days, *i.r*.) in these colitic mice was not able to modify the α-diversity when compared with vehicle-treated reactivated mice (Figures 7A,B). Using unweighted β-diversity, we observed that bacterial communities both from fecal and mucosa-associated microbiota (MAM) samples collected from DSS mice clustered separately from non-colitic mice (*P* < 0.05). However, hCTS treatment in colitic mice did not change the bacterial composition in the colitic mice (Figures 8A,B). We also assessed the effect of hCTS on colitic mice in the context of abundant phyla (percentage within the community >1%) and we observed no significant effect of hCTS treatment on the abundant phyla of colitic mice both in fecal and MAM samples (Figures 9A,B).

## Discussion

The mucosa plays a major role as a first-line of defense against environmental antigen exposure in the gastrointestinal tract (69). This mechanism of defense is aided by various antimicrobial peptides derived from the endocrine cells such as EC cells, which reside in the epithelium (70), and one of these gut antimicrobial peptides, which is hCTS (71, 72). This study, for the first time, demonstrates the anti-inflammatory effect of hCTS (1.5 mg/kg/day, *i.r*., 7 days) during reactivation of colitis using a quiescent model of colitis, which mimics the natural history of UC. We observed that treatment with the peptide downregulated the macroscopic score and/or histological score and also downregulated the M1 macrophages and the pro-inflammatory cytokine secretion. However, the peptide neither affected the M2 macrophages and the anti-inflammatory cytokine secretion in the colon nor the gut microbial dysbiosis observed in colitic mice.

During gut inflammation, macrophages infiltrate gut tissue in response to pro-inflammatory proteins (38). These macrophages possess distinct characteristics compared to their tissue resident macrophages counterparts and are classified as M1 macrophages (38, 73). In our study, gene expression revealed that hCTS treatment is associated with a downregulation of *iNOS* and *Mcp1* M1 macrophages markers during reactivation of colitis. Several studies confirming the elevation of iNOS and MCP1 in IBD patients (74–78) as well as experimental colitis models (79, 80) and constitutive expression of iNOS worsen UC symptoms by direct cytotoxicity, neutrophil activation (81), widening blood vessels (82), and/or elevating nitrosamines production (83). In addition, peroxynitrite, which activates poly-ADP ribosyl synthetase, results in cellular injury during gut inflammation *via* epithelial cell apoptosis and is assumed to be increased *via* iNOS upregulation (84, 85). In experimental colitis, systemic administration of specific iNOS inhibitor such as amino-guanidine or *N*-(3-(aminomethyl)benzyl) acetamidine results in amelioration of colitic symptoms, which further validates the pro-inflammatory effect of iNOS (86, 87). Concurrently, it is suggested that MCP1 acts as a chemoattractant by increasing the expression of integrins resulting in an increased immune cells infiltration. This statement was further validated using *Mcp1* knock-out mice (*Mcp1*^−/−^), where it has been observed that dinitrobenzene sulfonic acid-induced colitis in *Mcp1*^−/−^ mice resulted in downregulation of colonic inflammation compared to their WT counterparts, which were associated with a lower number of classical macrophages and CD3^+^ cells in their colon when compared to WT colitic mice (88). Thus, the decrease expression of *iNOS* and *Mcp1* following hCTS administration in our chronic colitic condition might explain the abrogation of the inflammation. Conversely, M2 tissue-resident macrophages are hyporesponsive to pro-inflammatory stimuli (73), and this population is crucial to control impaired activation of inflammation against commensal microbes; in this manner, the downstream signaling of TLRs and/or NF-κβ activation is abrogated (73, 89, 90). These M2 macrophages produce IL-10, which maintains the quiescent state of macrophages in the intestine. Moreover, they produce TGF- β, which also downregulates TLR signaling in blood monocytes, which is important to maintain a steady-state immune environment in laminal propria (73). Several studies confirm that defective in M2 macrophage polarization might increase the colitis severity in experimental models (91, 92). M2 macrophages in IBD patients also have defective functioning, which is supported by an *in vitro* study demonstrating that blood macrophages collected from CD patients and stimulated to M2 macrophages resulted in altered expression of CD40 and CD163 compared to healthy cells (93). Arg1 is classical M2 macrophage marker (94), and studies confirm lower *Arg1* mRNA expression in UC patients (95). The arginase enzyme (ARG1) compete with inducible nitric oxide synthase (iNOS) for l-arginine (l-ARG) as a substrate. The ARG1 metabolizes l-ARG to urea and l-Ornithine, the latter is important for cell division and collagen synthesis (96). Conversely, iNOS metabolizes l-ARG to l-Citrulline and nitric oxide which ultimately disrupts lipid, proteins, and DNA *via* producing peroxynitrites and/or hydrogen peroxide (96). In our study, although hCTS treatment did not have a direct effect on *Arg1* expression but the treatment might provide a competitive advantage toward l-ARG for ARG1 enzyme *via* downregulating *iNOS* expression. Concurrently, *Ym1*, another classical M2 macrophage marker (94) was not also affected by hCTS treatment. Although the functional role of Ym1 during inflammation continues to be studied, data suggest that it might help to regain immune homeostasis *via* modulating the toxic degradation effect of iNOS on heparin/heparin sulfate in an inflamed milieu (97). However, because Ym1 is secretory in nature and has an intrinsic ability to form crystals, overexpression of Ym1 might be associated with fatal tissue injury. For example, in genetically modified mice such as SHP-1 (protein tyrosine phosphate)- or CD-40-L-deficient mice, hyperactive macrophages overexpress Ym1, which results in eosinophilic crystal formation in the lung (98). Thus, regulation of Ym1 expression seems crucial to maintain the physiological state. Although suppression of the M2 macrophage population aggravates DSS colitis, the significance of this macrophage population in UC is controversial. Although not confirmed yet, it is hypothesized that classical (M1) rather than anti-inflammatory macrophages (M2) are important for UC. This hypothesis is supported by granuloma formation and fibrosis that is observed more in CD rather than UC (38). The granulomas are formed because of defective bacterial clearance by tissue-resident macrophages and fibrosis that occurs because of excessive wound healing by M2 macrophage (38). Thus, our observed hCTS effect on M1 macrophage marker and no effect on M2 macrophage marker *in vivo* might be beneficial to maintain the homeostatic environment in the gut.

To further validate our *in vivo* observation related to the anti-inflammatory effect of hCTS exerted on the M1 macrophage population, we conducted *in vitro* experiments where we collected naïve peritoneum macrophages and polarized toward either an M1 or M2 macrophage profile in the presence or absence of hCTS. In the presence of the peptide, we observed a significant decrease of M1 macrophage markers (*iNOS, Mcp1*) expression associated with a significant decrease of pro-inflammatory cytokines (*Il6*, *Il1b*, and *Tnfa*) expression and their release in the medium. However, hCTS did not modify the expression of M2 polarized macrophage markers (*Arg1 and Ym1*) or the expression of anti-inflammatory cytokines (*Il10* and *Tgfb*). These observations support the notion that hCTS exerts its anti-inflammatory effect through the downregulation of not only the pro-inflammatory cytokine secretion but also through the regulation of M1 macrophage markers, without modifying the M2 population. In our previous study, we demonstrated that in the context of acute colitis and *in vitro*, hCTS treatment was associated with an increased phosphorylation of signal transducer and activator of transcription 3 (STAT-3) and inhibition of STAT-3 phosphorylation abrogated the observed anti-inflammatory effect (31). STAT-3 phosphorylation is important to induce the anti-inflammatory response in the host to regulate aggravated inflammation in a yet undefined mechanism (99). The anti-inflammatory effect of STAT3 also corroborates with mice studies observing that mice having STAT3-deficient macrophages produce excessive cytokine and develop colitis (100, 101). In addition, mice lacking STAT3 in endothelial cells also become extremely sensitive to LPS challenge (102). Besides our lab, another group has confirmed that *in vitro* hCTS treatment stimulates STAT-3 activation in adipose tissue to derive from diet-induced obese mice (103). Thus, we can hypothesize that the currently observed anti-inflammatory effect of hCTS seen during the reactivation of colitis occurs through the downregulation of the M1 population and potentially through the activation of STAT-3 phosphorylation. Further confirmatory studies are required.

Gut microbiota represents the largest amount of microbes present in mammals compared to any other mammalian tissue (39). Maintenance of healthy intestinal tissue environment largely depends on the balanced composition of commensal and pathobionts known as gut homeostasis. Alteration of the gut microbial homeostasis is known as gut dysbiosis and it is evident in UC patients (68). Recently, we observed that acute colitis induced *via* DSS resulted in gut microbiota dysbiosis, which was more prominent in fecal samples compared to colonic mucosa-associated (MAM) samples (44). In our study, we demonstrated that reactivation of quiescent colitis resulted in a distinct microbial composition when compared to control, both in fecal and MAM samples. This observation is supported by previous findings demonstrating a microbial dysbiosis in DSS-induced colitis in dogs (104) and rats (105). All these observations, along with our current study confirms that DSS strongly disrupts the gut microbial homeostasis both in acute and in the chronic colitic condition. However, whether the gut dysbiosis is causal or an outcome of gut inflammation in IBD and in colitic mice is remains controversial. hCTS is a well-known antimicrobial peptide (24, 71, 72), and our recent study demonstrated that administration of a 5-day regime of hCTS to naïve mice altered the gut microbial composition when compared to control, and it was associated with a prominent effect in feces rather than in MAM samples (45). In these naïve mice fecal samples, hCTS treatment increased the relative abundance of Bacteroidetes compared to Firmicutes, which was the opposite of results observed in acute DSS colitic mice fecal samples (44, 45). Because our previous study did not test the effect of hCTS on gut microbiota during the development of acute colitis; in our current study, we deciphered the effect of hCTS treatment on gut microbiota composition in the context of reactivation of quiescent colitis. Surprisingly, a 7-day regime of hCTS administration did not change the microbial composition compared to vehicle-treated colitic mice. This is likely because of the long-term dysbiosis induced by the three cycles of DSS on gut microbiota, which dominates the effect of hCTS. Prophylactic hCTS treatment in the acute colitic condition or long-term hCTS treatment in the chronic colitic condition and its effect on gut microbiota need to be determined.

Finally, it is well established that environmental antigen exposure in IBD patients changes the regulatory mechanisms leading to pathological features such as gut inflammation (106, 107). It is hypothesized that inflammation results in neuroendocrine system activation and EC cell hyperplasia, which in turn elevates serum ChgA (30, 108). Although the possible impact of elevated ChgA in gut inflammation remains unknown, our group for the first time, showed that hCTS derived from the distal portion of ChgA is increased both in IBD and experimental colitis mouse models (31). Thus, we confirmed a prophylactic anti-inflammatory effect of hCTS in acute ulceration (31). A major drawback of this previous observation was the acute murine model of colitis used. Because IBD is known to be a chronic condition associated with several flares, our previous acute experimental colitis model failed to mimic it. In this study, we overcame this challenge by developing a quiescence colitis model. Using this model, we further validated the prophylactic anti-inflammatory effect of hCTS observed in acute colitic mice and transposed it to a model mimicking the natural history of the disease. Because therapeutic treatments are required for IBD patients, this peptide could be a new therapeutic target. However, further confirmatory studies are required.

## Ethics Statement

The murine experimental protocol was approved by the University of Manitoba Animal Ethics Committee (15-010) and the research was conducted according to the Canadian Guidelines for Animal Research (46, 47).

## Author Contributions

MR and JEG conceived and designed the study. MR, NE, PM, LK, and OE conducted the experiments and performed the analyses. MFR interpreted the data and wrote the manuscript. EK, CB, and JEG reviewed the draft manuscript.

## Conflict of Interest Statement

The authors declare that the research was conducted in the absence of any commercial or financial relationships that could be construed as a potential conflict of interest. The reviewer, KL, and handling editor declared their shared affiliation, and the handling editor states that the process met the standards of a fair and objective review.
